# Influence of Obesity on Pneumococcus Infection Risk in the Elderly

**DOI:** 10.3389/fendo.2019.00071

**Published:** 2019-02-13

**Authors:** Daniela Frasca, Janet McElhaney

**Affiliations:** ^1^Department of Microbiology and Immunology, University of Miami Miller School of Medicine, Miami, FL, United States; ^2^Health Sciences North Research Institute, Sudbury, ON, Canada

**Keywords:** obesity, aging, inflammation, respiratory tract infections, pneumococcus

## Abstract

Obesity negatively affects immune function and host defense mechanisms. Obesity is associated with chronic activation of the innate immune system and consequent local and systemic inflammation which contribute to pathologic conditions such as type-2 diabetes mellitus, cancer, psoriasis, atherosclerosis, and inflammatory bowel disease. Individuals with obesity have increased susceptibility to contract viral, bacterial, and fungal infections and respond sub-optimally to vaccination. In this review, we summarize research findings on the effects of obesity on immune responses to respiratory tract infections (RTI), focusing on *Streptococcus pneumoniae* (“pneumococcus”) infection, which is a major cause of morbidity and mortality in the US, causing community-acquired infections such as pneumonia, otitis media and meningitis. We show that the risk of infection is higher in elderly individuals and also in individuals of certain ethnic groups, although in a few reports obesity has been associated with better survival of individuals admitted to hospital with pneumococcus infection, a phenomenon known as “obesity paradox.” We discuss factors that are associated with increased risk of pneumococcal infection, such as recent infection with RTI, chronic medical conditions, and immunosuppressive medications.

## Introduction

The increase in the frequency of obesity is a worldwide phenomenon. Obesity is defined as a body-mass index (BMI) ≥ 30 kg/m^2^, by both the Centers for Disease Control and Prevention (https://www.cdc.gov/obesity/adult/defining.html) and the World Health Organization (https://www.who.int/topics/obesity/en/) and is associated with several debilitating chronic diseases including cardiovascular disease (1), type-2 diabetes mellitus (T2DM) (2–4), cancer (5), psoriasis (6), atherosclerosis (7), and inflammatory bowel disease (IBD) (8). Published data indicate that high BMI negatively correlates with protective immune responses and obese individuals are highly susceptible to viral, bacterial, and fungal infections (9–11). Obesity also increases the risk of musculoskeletal disorders and chronic back/lower limb pain (12); reduces cognitive function and is considered a potential risk factor for Alzheimer's disease and dementia (13, 14); induces ovulatory infertility (15); increases the risk of early and late miscarriage, gestational diabetes and preeclampsia, and complicates labor and delivery (16); impairs respiratory function by reducing lung expansion and narrowing airways in the lung (17), leading to asthma (18), and obstructive sleep apnea (19). In general, obesity decreases both the healthspan and lifespan, increases premature mortality and significantly increases global healthcare costs. This global obesity epidemic affects all age groups as shown in a recent survey conducted on 68 million people from 195 countries (20).

Obesity negatively affects immune function and host defense mechanisms. One of the reasons is because obesity is an inflammatory condition associated with chronic activation of the immune system and consequent local and systemic inflammation which are negatively associated with a functional immune system. It has previously been shown that systemic chronic inflammation induces intrinsic inflammation in immune cells and a status of immune activation associated with reduced immune responses. Elevated serum levels of TNF-α typical of old age negatively correlate with T cell function, due to the down-regulation of CD28 gene transcription and cell surface expression (21). B cells are also affected by inflammation. We have shown that B cells from elderly individuals spontaneously make higher amounts of TNF-α than those from young individuals (22). B cell intrinsic TNF-α levels are positively correlated with serum TNF-α and, more importantly, these B cell levels of TNF-α before stimulation are negatively correlated with the function of the same B cells after *in vivo* or *in vitro* stimulation with the influenza vaccine or with mitogens, respectively. These findings are supported by the observation that inhibition of TNF-α improves both T (23, 24) and B cell (22) function.

The adipose tissue (AT) is a major immunological tissue that contributes to systemic inflammation. AT inflammation is characterized by infiltration and activation of immune cells secreting pro-inflammatory mediators, such as cytokines and chemokines, as well as adipokines, which recruit immune cells to the obese AT. Recruited immune cells differentiate into inflammatory subsets and secrete additional pro-inflammatory molecules which contribute to the maintenance of local and systemic inflammation. Immune cells infiltrating the AT include neutrophils, macrophages, T cells, B1 and B2 cells, NK cells, and innate lymphoid cells. The cellular composition of AT is dynamic and is regulated by acute and chronic stimuli including diet, body weight, and fasting.

Aging is associated with a progressive decline in physiological functions, leading to overt chronic disease, frailty and mortality. Physiological changes include inflammation of the AT, which leads to AT dysfunction, increased secretion of pro-inflammatory mediators, immune cell infiltration and accumulation of senescent cells. These processes altogether promote low-grade chronic inflammation [inflammaging (25, 26)] and insulin resistance, and lead to transition from metabolically normal obesity to metabolic syndrome. This occurs through metaflammation (27) in which excess nutrients, due to inefficient glucose metabolism, promote chronic low-grade inflammation. Metabolic hallmarks of metaflammation are high levels of glucose, lipids, free fatty acids, and reactive oxygen species. AT dysfunction may be a fundamental contributor to the elevated risk of chronic disease, disability, and adverse health outcomes in the elderly.

This review will show the experimental evidence that obesity is linked to higher severity of RTI in individuals of different ages similar to what has been shown in older adults. Potential mechanisms responsible for these effects will be discussed. We will focus primarily on *Streptococcus pneumoniae* (“pneumococcus”) infection, which is a major cause of morbidity and mortality in the US, causing community-acquired infections such as pneumonia, otitis media, and meningitis.

## Obesity and RTI

**Figure 1:**
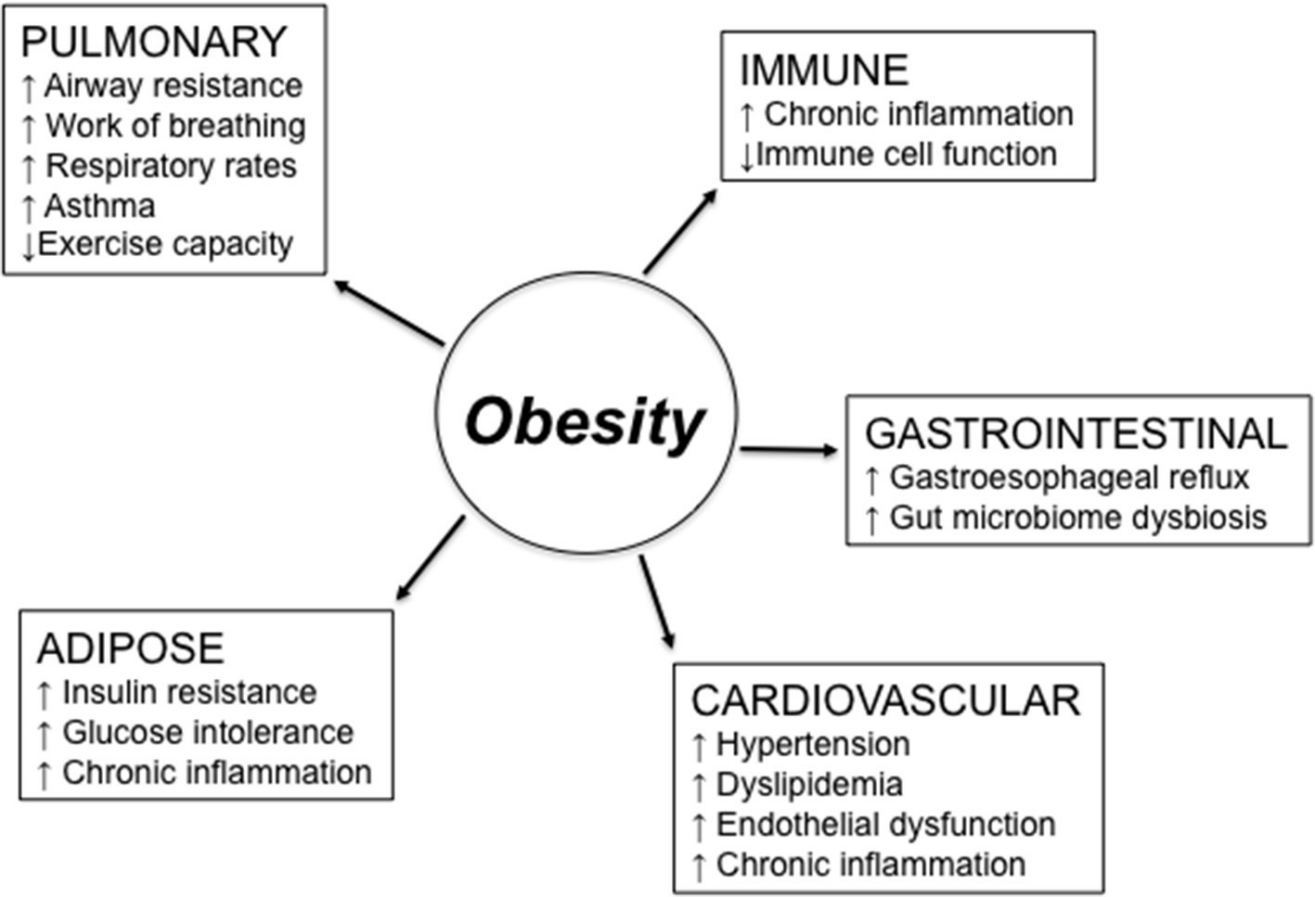
Obesity effects on different body systems involved in the response to respiratory tract infections. Obesity-induced changes in the lung may be due to higher weight load on the thorax and lead to reduced lung function. Obesity-induced changes in the immune system lead to chronic inflammation, immune activation, and reduced clearance of pathogens. Obesity also causes gastroesophageal reflux, which is a risk factor for aspiration pneumonia and asthma, due to the excess of belly fat posing pressure on the stomach, and gut microbiome dysbiosis. Cardiovascular complications of obesity include hypertension, dyslipidemia, and endothelial dysfunction. Obesity-induced changes in the adipose tissue are responsible for increased insulin resistance and glucose intolerance, both leading to chronic inflammation.

Figure 1 summarizes major obesity-associated changes in body systems which may be responsible for reduced responses to RTI. Lung function is altered in obesity (28). Altered lung mechanics and increased airway resistance related to obesity cause an increase in work of breathing and decreased exercise capacity. Thus, increased respiratory rates and complaints of fatigue are experienced by obese vs. lean individuals. These changes in lung function are mainly due to the higher weight load on the thorax, which is independent of any underlying parenchymal lung disease, significantly contribute to physical disability and impaired quality of life. Obese individuals with chronic obstructive pulmonary disease (COPD), a leading cause of morbidity and mortality worldwide, as well as with chronic bronchitis and emphysema, require increased muscular effort needed for ventilation and exhibit greater dyspnea (29, 30). Moreover, the sedentary lifestyle of these patients leads to increased fat accumulation in the lung and consequent airway obstruction.

In general, mechanisms related to the obesity-driven, low-grade chronic inflammation may be dramatically influenced by the presence of comorbid conditions. Obesity is in fact a complex metabolic condition associated with changes in many different physiological functions of the organism, and these changes, alone or in combination, may induce, support, and exacerbate lung inflammation. These conditions include gastroesophageal reflux, a risk factor for aspiration pneumonia and asthma (31), which may be worsened by hypertension and dyslipidemia, two conditions affecting immune responses in the lungs and increasing susceptibility to asthma and aspiration pneumonia (32).

Several pro-inflammatory cytokines and adipokines are secreted by the AT. These mediators, released into the circulation, contribute to the low-grade chronic inflammation (33) and induce pulmonary inflammation. The lung is continuously exposed to insults from the air and also to toxic molecules circulating through the pulmonary and bronchial vasculature. Mouse studies have shown that different molecules, such as bacteria, ozone, allergens, and particulate matter, activate the AT to secrete pro-inflammatory mediators involved in the generation of pulmonary inflammation (34–37). In addition, mechanisms of defense against pathogens and smaller particles that have successfully penetrated the mucosal barrier, including first-line filtration, mucosal IgA, alveolar cells, and resident immune cells in the parenchyma are decreased in obese individuals leading to low-grade chronic inflammation.

Leptin is an adipokine primarily secreted by the AT (38). Its effects on systemic and pulmonary inflammation in the settings of obesity have been the focus of several studies. Plasma levels of leptin positively correlate with the amount of body fat and BMI, increase with age (39), and contribute to the inflammatory status of the AT associated with obesity. Leptin induces the secretion of inflammatory cytokines by macrophages (40), T cells (41), and B cells (42, 43) *in vitro*. Leptin has also been reported to increase leukotriene synthesis by alveolar macrophages, leading to pulmonary inflammation (44). Leptin is known to down-regulate functional immune responses (45). There is evidence that cells in the lung may also be capable of secreting leptin (46, 47), although it seems more likely that leptin is found in the lung as a consequence of increased microvascular permeability associated with lung inflammation (48).

### Viral RTI

Viruses causing severe RTI in elderly individuals include influenza A and B virus, respiratory syncytial virus (RSV), human parainfluenza virus (HPIVs), rhinovirus, enterovirus, human coronavirus (HCoVs). The effects of obesity have only been described for a few of these viral infections.

While many studies have investigated the effect of obesity or aging on the risk of influenza virus, only a few studies have analyzed the effect of both, likely because obesity has been shown to induce defects in peripheral immune cells similar to those induced by aging. We hypothesize that multifactorial age-associated conditions and parameters might be concomitantly associated with the predisposition of older adults to be infected with the influenza virus. During the 2009 influenza pandemic season, it was shown that obesity was positively associated with reduced pulmonary immune defenses not only against the Influenza A(H1N1)pdm09 virus but also against other pathogens (49). Several reports have clearly indicated that obese and morbidly obese individuals were more susceptible to infection with the Influenza A(H1N1)pdm09 virus, to a greater severity of illness after infection (49–51), to higher rates of hospitalization (50), admission to intensive care units (52), and death not only in the US (49) but also in many other countries (53–56).

The importance of RSV is increasingly recognized in hospitalized adults, but mainly in those 65 years and older. Vaccines for the prevention of RSV infections are not yet available, and development efforts are made more difficult in the older population due to age-associated decreases in immune responses. RSV infection in older adults causes great suffering due to hospitalization and death and is considered a social burden similar to that of seasonal influenza (57, 58). Clinical manifestations of RSV infections are similar to those caused by other viral respiratory pathogens. Most of the studies published on RSV-associated hospitalizations have been conducted in individuals ≥65 years, with 5–10% of hospitalizations for acute respiratory illnesses due to RSV infection. Older adults with COPD and/or congestive heart failure have been shown to be at higher risk (57, 59). A study conducted in middle-aged and older adults has shown that RSV infection was associated with obesity (60). Although there are no published studies on RSV infection in obese individuals, we can hypothesize that the clinical effects are similar to those observed in older adults.

### The Bacterial RTI With *Streptococcus Pneumoniae*

Infection with the gram-positive bacteria *Streptococcus pneumoniae* (“pneumococcus”), a common pathogen in the nasopharynx most commonly associated with pneumonia, represents a major cause of morbidity and mortality. The risk of infection is higher in obese vs. lean individuals. Obesity has been associated with increased risk of pneumonia in young individuals (61). Studies on the effect of pneumococcus infection in obese elderly individuals are limited, but one study has reported that the incidence of community-acquired pneumonia in obese patients is directly associated with higher BMI in both age groups (62). However, other studies have conversely shown that obese compared to lean individuals are 2-fold more likely to survive after being admitted to hospital with pneumococcus infection, suggesting that extra energy may help to fight both infection and inflammation (63). After adjustment for potential confounders, morbid obesity was not associated with mortality, whereas obesity was associated with decreased mortality. Neither morbid obesity nor obesity were associated with admission into intensive care units and use of mechanical ventilation. This apparently controversial result may be due to the fact that when BMI is used as a measure of adiposity results may differ across different study populations. BMI is a crude anthropometric biomarker and it does not take into account different important measures of adiposity, such as fat mass, body fat distribution, measures of central adiposity, and nutritional status. Another reason may be due to the different inflammatory profile of the participants recruited into the studies. For example, obese individuals with high circulating levels of leptin, the adipokine secreted in large amounts during obesity, may have enhanced local immune responses against respiratory pathogens and increased host defense mechanisms in the lung (64). Leptin is a strong immunomodulator of both innate and adaptive immune responses and increases macrophage phagocytosis, neutrophils chemotaxis and natural killer cell cytotoxicity, as well as B and T cell function, leading to increased bacterial clearance. Therefore, increased leptin levels could increase immune responses of obese individuals and better protection against infection. Although a recent study of survivors of community-acquired pneumonia showed that this obesity paradox could not be attributed to differences in biomarkers of several inflammatory pathways (65), this study has only measured four markers of inflammation (not including leptin) and did not distinguish pneumonia from other causes of death, limiting the conclusions about inflammation as the pathophysiological explanation of the obesity paradox.

The risk of infection is also significantly higher in individuals aged 65 years and older as compared to younger individuals (66). Table 1 summarizes major studies cited in this review showing the effects of age and comorbidities on mortality rates after infection with pneumococcus. Before the availability of antimicrobial treatments, >70% of patients hospitalized died of bacterial pneumococcal pneumonia and mortality rates were even higher in older adults (73). By the end of the twentieth century, mortality rates had dropped to 20% in individuals ≥65 years of age and to 40% in those ≥85 years of age (67–69). The American Centers for Disease Control and Prevention (CDC) reported that in 1995, in 4 areas of the US, the rate of invasive pneumococcal infection among older adults was 3-fold higher than infections with group B streptococcus, 10-fold higher than with *Haemophilus influenzae*, and 25-fold higher than meningococcus or *Listeria monocytogenes* infections (66). Rates of infection in elderly individuals from Asia, Africa or South America are less known.

**Table 1:** Effect of age and comorbidities on mortality rates after pneumococcus infection.

**Population age**	**Comorbidities**	**Mortality rates**	**Reference**
18–64 years	None reported^a^	19%	(67)
≥64	T2DM^b^	45%	(68)
	Chronic lung disease	33%	(68)
	Congestive heart failure	20%	(68)
	Chronic renal failure	60%	(68)
≥65	None reported	20%	(66)
≥65	AIDS^c^	69%^e^	(69)
	SLE^d^	(All comorbidities)	(69)
	Chronic lung disease		(69)
	Chronic liver disease		(69)
	Congestive heart failure		(69)
	Chronic renal failure		(69)
80	None reported^a^	71%	(70)
≥85	None reported^a^	38%	(67)
78–100 years	None reported^a^	27%	(71)
86–104 years	None reported^a^	20%	(72)

a *This study analyzed the total population, including healthy, and non-healthy individuals. The mortality rates due to the different comorbidities are not reported*.

b *T2DM, type-2 diabetes mellitus*.

c *AIDS, acquired immune deficiency syndrome*.

d *SLE, systemic lupus erythematosus*.

e *In this study mortality rates were calculated considering the total population (healthy and not healthy with the listed comorbidities)*.

In addition to obesity and aging, several other risk factors have been identified, including previous viral RTI such as influenza and RSV (74–76) as well as chronic illnesses such as COPD, congestive heart failure, cerebrovascular diseases and dementia, cancer, and T2DM (77). Use of corticosteroids has also been shown to be significantly associated with the risk of pneumococcus infection (77).

The incidence of pneumococcal pneumonia increases with age and the number of co-morbidities; those with 2 at-risk conditions have a similar risk to those with a high-risk rheumatologic condition, and those with ≥3 co-morbidities have a 2-fold higher risk compared to those with a rheumatoid condition (78). The age-associated increase in low-grade chronic inflammation has been shown to be associated with increased susceptibility to pneumococcal infection, with higher disease severity and decreased survival in older adults (79, 80). In general, microbial dysbiosis drives intestinal permeability and translocation of bacterial components into the bloodstream, further sustaining inflammation, immune activation, and decreased immune responses (81). Increased gut permeability with age induces not only systemic but also lung inflammation and tissue damage, as shown by increased levels of circulating bacterial toxins, leading to pulmonary endothelial damage.

Not only the gut microbiota, but also the upper RT (URT) (82) microbiota changes with age contributing to *Streptococcus pneumoniae* colonization and its inefficient clearance, as shown by studies conducted in mice (83). The URT is colonized by several different species of pathogens and is continuously exposed to bacteria present in the environment, which survive in the nasal and oral cavities of older individuals, due to loss of resistance to colonization and altered immunity. It has been shown that efficacy of intranasal vaccination with a live attenuated influenza virus, measured by mucosal IgA secretion, depends on the specific bacterial composition of the nasal cavity, suggesting the importance of nasal microbiota for nasal immunity (84). Whether the URT microbiota of obese individuals contributes to *Streptococcus pneumoniae* colonization remains to be investigated by further studies.

Obesity is associated with changes in gut microbiota at phylum-level and with reduced bacterial diversity in mice and humans (85, 86). Mouse studies have identified intestinal microbiota products that protect the host from pneumococcus infection and have shown the mechanisms involved. Briefly, it was shown that the gut microbiota increases phagocytosis of alveolar macrophages and protects from tissue damage during pneumococcus-induced sepsis (87). Human studies are necessary to confirm the positive results obtained in mice.

Nursing homes represent one of the settings for outbreaks of pneumococcal infection in the elderly. Vaccination has been reported to protect < 10% of elderly individuals in nursing homes during outbreaks of pneumococcal infection (70–72).

The risk of pneumococcal infection is also higher in individuals of certain ethnic groups. Afro-American people of all ages living in the US are 2- to 4-fold more susceptible than Caucasian individuals, but rates of infection are only slightly higher in the older Afro-American population (88, 89). Native Americans and Alaskans are at higher risk of pneumococcal disease than individuals of other ethnic groups. In the population ≥60, Native Alaskans as well as Native Americans of the Apache tribe living in Arizona had a 2-fold increase risk of pneumococcal disease as compared to non-native populations living in the same area (90, 91). In Northern Canada as well, higher risk of pneumococcal disease has been reported for Indigenous populations (92). Higher rates of multiple chronic conditions related to the colonization of Indigenous people's diets over many generations may, in part, explain these disparities, and require further study (93).

## Conclusions

Chronic activation of the innate immune system and consequent local and systemic inflammation related to obesity contributes to pathologic conditions such as T2DM, cancer, atherosclerosis, and IBD. While obese individuals have increased susceptibility to viral, bacterial and fungal infections, outcomes of these infections may be determined by a host of other factors. This is evident when comparing outcomes of influenza and pneumococcal pneumonia; while both older age and obesity contribute to the serious complications of influenza, obesity appears to be protective against the serious complications of pneumococcal pneumonia with advancing age, the so-called “obesity paradox.” Future studies will need to address the significant gaps in understanding the interaction of age, obesity, multiple chronic conditions, and the microbiome, particularly related to the risk for and complications of acute RTI. Mechanistic studies are needed to move beyond what has been learned from epidemiologic studies to develop new biomarkers and related preventive and therapeutic approaches to improving outcomes of acute respiratory illness in persons with multiple chronic conditions. Future studies will also need to address prevention of obesity, by improving eating habits and increasing physical activity, as a way to fight RTI. There is indeed experimental evidence showing that weight reduction decreases systemic inflammation and improves immune responses against bacterial, viral and fungal infections. Several epidemiological studies have evaluated the effects of diet and exercise in protecting subjects from several diseases associated with chronic low-grade inflammation. This will reduce the risk for infectious diseases, will increase their responses to pathogens, and reduce the burden of illness and health-related costs.

## Author Contributions

All authors listed have made a substantial, direct and intellectual contribution to the work, and approved it for publication.

### Conflict of Interest Statement

JM has received honoraria from Sanofi, GSK and Pfizer for participation in advisory boards and scientific presentations at meetings, and related costs of travel. The remaining author declares that the research was conducted in the absence of any commercial or financial relationships that could be construed as a potential conflict of interest.
